# Evaluation of a genetically modified foot-and-mouth disease virus vaccine candidate generated by reverse genetics

**DOI:** 10.1186/1746-6148-8-57

**Published:** 2012-05-16

**Authors:** Pinghua Li, Xingwen Bai, Pu Sun, Dong Li, Zengjun Lu, Yimei Cao, Yuanfang Fu, Huifang Bao, Yingli Chen, Baoxia Xie, Zaixin Liu

**Affiliations:** 1 State Key Laboratory of Veterinary Etiological Biology, National Foot and Mouth Disease Reference Laboratory, Key Laboratory of Animal Virology of Ministry of Agriculture, Lanzhou Veterinary Research Institute, Chinese Academy of Agricultural Sciences, Lanzhou, 730046, Gansu, China

## Abstract

**Background:**

Foot-and-mouth disease (FMD) is the most economically important and highly contagious disease of cloven-hoofed animals worldwide. Control of the disease has been mainly based on large-scale vaccinations with whole-virus inactivated vaccines. In recent years, a series of outbreaks of type O FMD occurred in China (including Chinese Taipei, Chinese Hong Kong) posed a tremendous threat to Chinese animal husbandry. Its causative agent, type O FMDV, has evolved into three topotypes (East–South Asia (ME-SA), Southeast Asia (SEA), Cathay (CHY)) in these regions, which represents an important obstacle to disease control. The available FMD vaccine in China shows generally good protection against ME-SA and SEA topotype viruses infection, but affords insufficient protection against some variants of the CHY topotype. Therefore, the choice of a new vaccine strain is of fundamental importance.

**Results:**

The present study describes the generation of a full-length infectious cDNA clone of FMDV vaccine strain and a genetically modified virus with some amino acid substitutions in antigenic sites 1, 3, and 4, based on the established infectious clone. The recombinant viruses had similar growth properties to the wild O/HN/CHA/93 virus. All swine immunized with inactivated vaccine prepared from the O/HN/CHA/93 were fully protected from challenge with the viruses of ME-SA and SEA topotypes and partially protected against challenge with the virus of CHY topotype at 28 days post-immunization. In contrast, the swine inoculated with the genetically modified vaccine were completely protected from the infection of viruses of the three topotypes.

**Conclusions:**

Some amino acid substitutions in the FMDV vaccine strain genome did not have an effect on the ability of viral replication in vitro. The vaccine prepared from genetically modified FMDV by reverse genetics significantly improved the protective efficacy to the variant of the CHY topotype, compared with the wild O/HN/CHA/93 virus. Thus, the full-length cDNA clone of FMDV can be a useful tool to develop genetically engineered FMDV vaccine candidates to help control porcinophilic FMD epidemics in China.

## Background

Foot-and-mouth disease (FMD) is a highly contagious vesicular disease of domestic and wild cloven-hooved animal species, which is caused by the foot-and-mouth disease virus (FMDV), the prototype member of the genus Aphthovirus of the family *Picornaviridae*. The highly contagious nature of FMDV and the associated high morbidity and productivity losses make it one of the most important barriers to the world trade of live animals and animal products. Control of the disease has been based on large-scale vaccinations with whole-virus inactivated vaccines, limitation of animal movements and destruction of herds exposed to the virus [1,2]. The currently available vaccine shows generally good protection against infection with the homologous and antigenically related viruses. However, difficulties facing the eradication of FMD include the antigenic diversity of FMDV in nature, which has been reflected in the identification of seven serotypes (A, O, C, SAT1, SAT2, SAT3 and Asia1) [3] and multitudes of antigenic variants that often co-circulate in a given geographical area [4]. The emergence of antigenically novel viruses, against which existing vaccines do not provide adequate protection, may require the selection of new vaccine strains to control the viruses circulating in the field.

The FMD virion consists of a single-stranded RNA genome packaged in an icosahedrally symmetric protein shell, which is composed of 60 copies each of four structural proteins 1A (VP4), 1B (VP2), 1 C (VP3) and 1D (VP1) [5]. Three of these proteins, VP1, VP2 and VP3, contribute to the formation of five known antigenic sites of type O FMDV [6,7]. The G-H loop and carboxy terminus of VP1 contribute to site 1, key residues have been shown to be 144, 148, 154 and 208, respectively. Amino acids at positions 31, 70–73, 75 and 77 of VP2 contribute to site 2. Site 3 involves the B-C loop of VP1, in which key residues have been shown to be 43 to 48 [6]. Only one critical residue, at position 58 of VP3, has been identified for site 4. Site 5 maps to a single residue (residue 149) on the G-H loop of VP1 that is distinct from site 1. While much of the antibody response to FMDV can be directed at the G-H loop of VP1, all of the sites appear to be necessary for a complete immunologic response to either infection or vaccination [8,9].

Among the seven serotypes of FMDV, serotype O is prevalent in China (including Chinese Taipei, Chinese Hong Kong) and its surrounding countries [10-13]. Recent studies have showed that type O FMDV in these region were clustered into three topotypes, namely Middle East–South Asia (ME-SA), Southeast Asia (SEA), and Cathay (CHY) [13,14]. An inactivated FMD vaccine prepared from O/HN/CHA/93 strain that belongs to the CHY topotype [15], is currently available in China. This vaccine is used to protect against type O FMD epidemics, which often provides complete protection against ME-SA and SEA topotype virus infection, but can’t affords good protection against some variants of the CHY topotype. Therefore, the development of new vaccine strains is of urgently needed. However, the development of useful cell-culture-adapted vaccine strains from field isolates is time-consuming and expensive, limiting the availability of custom-made vaccine strains [16]. The recent progress in animal RNA virus vaccine development, particularly the reverse genetics system-based for vaccine development [17-21], provided a perspective on potential novel strategies and approaches to develop a virus vaccine candidate.

The present study describes the generation of an infectious cDNA clone of FMDV O/HN/CHA/93 vaccine strain and a genetically modified virus with some amino acid substitutions in antigenic site 1 (VP1 134 C → S, 137 S → G, 139 A → T, 140 R → S, 142 V → T, 142 S → N), site 3 (VP1 43 T → K, 48 I → V), and site 4 (VP3 58 E → D), based on the established infectious clone. The replication kinetics in vitro of the recombinant viruses and the protective efficacy of inactivated oil-emulsified vaccines prepared from the genetically modified virus and the wild O/HN/CHA/93 virus against isolates of three topotypes were evaluated.

## Methods

### Analysis of amino acid variation of isolates of CHY topotype

**Figure 1:**
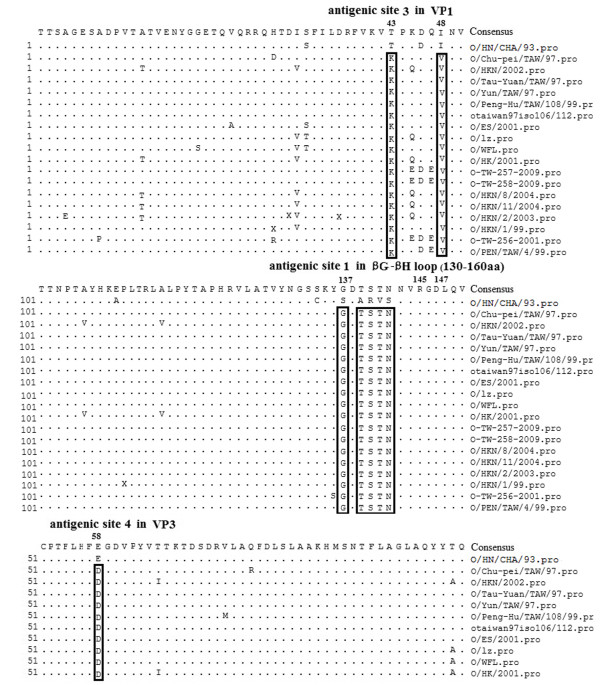
**Alignment of amino acid sequences of O/HN/CHA/93 vaccine strain and 18 reference viruses.** Only sequences different from the consensus are shown. Sequence data of isolates in the present work are obtained from the GenBank.

The P1 or VP1 sequence data of 18 FMD reference isolates were obtained from GenBank. O/HN/CHA/93 shared high homology with O/GD/China/86 (GenBank AJ131468) [22]. The deduced amino acid sequence alignments were prepared using the data for each FMDV using SeqMan II (DNAStar Lasergene 8.0). In brief, the amino acid sequences of P1 or VP1 were maximally aligned using the MegAlign program. All the reference isolates were clustered into the CHY topotype [11,12,23-25]. Table 1 shows the origin of all reference isolates characterized in the present study and Figure 1 shows the analysis of amino acid sequences of vaccine strain and reference strains.

**Table 1:** The origin of type O foot-and-mouth disease viruses characterized in the present study

**Isolate**	**GenBank accession no.**	**Species**	**Place isolated**	**Date collected**
O/HN/CHA/93	AJ131468	Porcine	HeNan	1993
O/Chu-pei/TAW/97	AF026168	Porcine	Taiwan	1997
O/HKN/2002	AY317098	Porcine	Hong Kong	2002
O/Tau-Yuan/TAW/97	AF154271	Porcine	Taiwan	1997
O/Yun/TAW/97	AF308157	Porcine	Taiwan	1997
O/Peng-Hu/TAW/108/99	AY593833	?	Taiwan	1999
otaiwan97 iso106/112	AY593835	?	Taiwan	1997
O/ES/2001	AY686687	?	?	2001
O/lz	DQ248888	?	?	?
O/WFL	EF175732	?	?	?
O/HK/2001	EU400597	Porcine	Hong Kong	2001
O-TW-257-2009	GQ292739	Porcine	Taiwan	2009
O-TW-258-2009	GQ292740	Porcine	Taiwan	2009
O/HKN/8/2004	DQ164885	Porcine	Hong Kong	2004
O/HKN/11/2004	DQ164888	Porcine	Hong Kong	2004
O/HKN/2/2003	DQ164879	Porcine	Hong Kong	2003
O/HKN/1/99	AJ294925	Porcine	Hong Kong	1999
O-TW-256-2001	GQ292738	Porcine	Taiwan	2001
O/PEN/TAW/4/99	AJ294928	Porcine	Taiwan	2001

Question mark (?) indicates that data are absent.

### Cell lines, viruses

Baby hamster kidney cell (BHK-21) [26] was maintained in Dulbecco’s Modified Eagle Medium (DMEM) supplemented with 10% fetal calf serum (FBS). BSR-T7/5 cells (a BHK derivative that stably expresses T7 RNA polymerase) [27], were maintained in Glasgow Minimal Essential Medium (GMEM) supplemented with 4% tryptose phosphate broth, 10% FBS, and in alternate passages geneticin was added to 1 mg/ml to ensure maintenance of the T7 polymerase gene.

FMDV O/HN/CHA/93 vaccine strain was used to construct the infectious clone and genetically modified mutant. O/HN/CHA/93 was adapted to grow in BHK cells for use in preparation of an inactivated vaccine to help control type O FMD epidemics in China. The O/Tibet/CHA/99, O/TAW/TL/97, and O/JX/CHA/2010 viruses used in the present study were obtained from the National FMD Reference Laboratory at Lanzhou Veterinary Research Institute. The O/Tibet/CHA/99 strain is of the ME-SA topotype [24], the O/TAW/TL/97 strain is of the CHY topotype [24], whereas the O/JX/CHA/2010 strain was originally isolated in China in 2010 and is of the SEA topotype [28]. Three viruses were titrated in BHK-21 cells.

### Construction of genome-length cDNA clone of O/HN/CHA/93 vaccine strain

**Figure 2:**
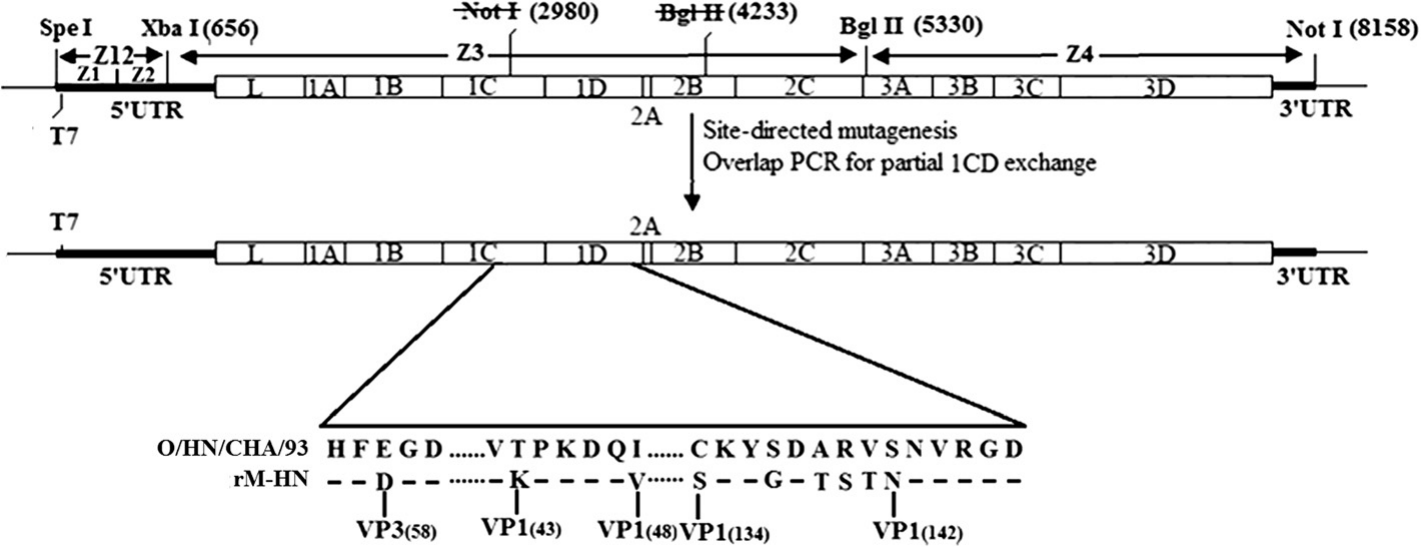
**Strategy used to construct FMDV O/HN/CHA/93 full-length cDNA clone and genetically modified clone.** The location of the restriction enzyme cleavage sites used to assemble the subcloned PCR fragments (Z1, Z2, Z3 and Z4) are shown (numbered relative to nucleotide position in the virus genome). Thick lines and an open box represent the untranslated regions and the open-reading frame for the viral polyprotein, respectively. The thin line represents the vector sequence. FMDV cDNA is under the control of the T7 promoter.

All the molecular constructs were prepared by using standard molecular biological techniques [29]. Total RNAs were extracted from the virus stock of O/HN/CHA/93 using RNeasy mini kit (Qiagen). Viral cDNAs were performed using M-MLV reverse transcriptase (Invitrogen) and specific RT primers. Four overlapping cDNA fragments (designated Z1-Z4 (Figure 2)), representing the entire viral RNA genome, were amplified by PCR using primer sets Z1/Z1′, Z2/Z2′, Z3/Z3′ and Z4/Z4′, respectively. Table 2 lists the primers used to perform RT-PCR for preparation of full-length cDNA clones of O/HN/CHA/93 virus. The Z1 primer contains a *Spe* I site, T7 promoter sequence and three nonviral G residues at the 5′ end of the FMDV genome, the Z4′ primer was engineered to add a *Not* I site following the viral poly(A) tail sequence. The PCRs were carried out using PrimeSTAR HS DNA Polymerase (Takara) to enhance the fidelity during the DNA synthesis. The Z1 and Z2 fragments were then fused by a second round of PCR with primers Z1 and Z2′ to generate Z12 fragment. Subsequently, the resulting amplicons was ligated into the pMD-20 vector, leading to the positive clone pMDZ12. The other two PCR products (Z3 and Z4) were separately cloned into the corresponding site of M-pSK vector [30], which is a derivative of pBluescriptSK (+) vector by removing T7 promoter sequence and modifying some restriction enzyme sites. The resulting positive plasmids were designated pSK-Z3 and pSK-Z4, respectively. Quick-Change®Multi Site-Directed Mutagenesis Kit (Stratagene) was used to introduce two silent mutations (GCG (Ala)-GCC (Ala), ATC (Ile)-ATA (Ile)) into Z3 segment to eliminate *Not* I (nucleotide position 2980) and one of *Bgl* II sites (nucleotide position 4233) (Figure 2), which will be as genetic tags by PCR amplification of the parental plasmid pSK-Z3 using Ztu1/Ztu1′ and Ztu2/Ztu2′ primer pairs, respectively. The modified plasmid named pSK-Z3M, was confirmed by complete DNA sequencing.

**Table 2:** Nucleotide sequence of PCR primers used for the construction of FMDV O/HN/CHA/93 full-length cDNA clone

**Name**	**Nucleotide Sequence (5′ → 3′)**	**Nucleotide Position (nt)**
Z1	AG**ACTAGT***taatacgactcactata*_*T7promoter*_GGGTTG	1–20
	AAAGGGGGCGCTAGGGT	
Z1′	GAGGAGGGGGGGGGGGGGGGTGAAAG	363–388
Z2	CTTTCACCCCCCCCCCCCCCCTCCTC	363–388
Z2′	GAAGCAACAGTGCTGCTACT	721–740
Z3	CGCGCCGTCGCTTGAGGAAG	560–579
Z3′	GGGTCGGAGCTCCTCCTTGATAGA	5433–5456
Z4	AGCCACCTCTTCAGAACGTCTACC	5242–5265
Z4′	AC**GCGGCCGC**TTTTTTTTTTTTTTTTTTTTT	3′ end
Ztu1	GGAGAGCGAGTCAGAT**A**TCTTCTTCACTACA	4221–4251
Ztu1′	TGTAGTGAAGAAGA**T**ATCTGACTCGCTCTCC	4221–4251
Ztu2	GAAAACGCCTGAGGC**C**GCCGCACACTGCATT	2967–2997
Ztu2′	AATGCAGTGTGCGGC**G**GCCTCAGGCGTTTTC	2967–2997

Restriction enzyme sites introduced during clone construction are boxed. Mutated nucleotides are underlined and in bold.

The full-length cDNA clone of O/HN/CHA/93 was constructed by the following ligations. First, the *Spe* I-*Xba* I fragment (Z12) derived from pMDZ12 was ligated into pSK-Z3M plasmid which was digested with the same enzymes. The resulting plasmid was named pSK-Z123. Then, the *Bgl* II-*Not* I fragment (Z4) derived from pSK-Z4 was cloned into *Bgl* II- and *Not* I-digested pSK-Z123 plasmid, leading to the construct pOFS, which containing the full-length genome of FMDV O/HN/CHA/93 vaccine strain. The full-length clone was fully sequenced to ensure that additional mutations had not been introduced during cloning. Figure 2 illustrates the strategy for the construction of the full-length cDNA clone.

### Construction of genetically modified FMDV full-length cDNA clone

For construction of genetically modified FMDV full-length cDNA clone, overlap PCR fusion were used to introduce six amino acids substitutions at G-H loop of VP1 (134 C → S, 137 S → G, 139 A → T, 140 R → S, 142 V → T, 142 S → N). In brief, individual parts were amplified with ZP1F/ZP3R and ZP2F/ZP4R primer pairs, respectively, and then these two fragments were fused by a second round of PCR with primers ZP1F and ZP4R. Consequently, the desired mutations were in the center of ZP2R and ZP3F primers. The resulting amplicon was then digested with *Bss*H II/*Xma* I and cloned into pSK-Z3M plasmid, which had been digested with the same enzymes, leading to the positive clone pSK-Z3MT. The correct nucleotides were confirmed by complete DNA sequencing. Then, the plasmid pSK-Z3MT was used as the backbone for the followings site-directed mutagenesis to produce amino acid substitutions (1 C 58 E → D, 1D 43 T → K, and 48 I → V) with HN2729F/HN2729R, HN3341F/HN3341R, and HN3356F/HN3356R primer pairs using Quick-Change®Multi Site-Directed Mutagenesis Kit, respectively. After three rounds of site-directed mutagenesis, the resulting clone named pSK-Z3MTΔ, was sequenced to confirm that the expected modifications had been introduced during amplification. Finally, the plasmid pSK-Z3MTΔ was digested with *Spe* I/*Bgl* II and cloned into the corresponding region of pOFS plasmid to produce genetically modified FMDV full-length cDNA clone pOFSM. The final modified construct was confirmed by complete DNA sequencing. Table 3 lists the primers used for site-directed mutagenesis and overlap PCR fusion.

**Table 3:** Nucleotide sequence of PCR primers used for the construction of genetically modified clone

**Name**	**Nucleotide Sequence(5′ → 3′)**	**Nucleotide Position (nt)**
HN2729F	TCCTACACTTC**GAC**GGTGACGTACCGTAC	2729–2752(E → D)
HN2729R	GTACGGTACGTCACC**GTC**GAAGTGTAGGA	2729–2752(E → D)
HN3341F	GATTTGTGAAAGTC**AAA**CCAAAAGACCAAAT	3341–3371(T → K)
HN3341R	ATTTGGTCTTTTGG**TTT**GACTTTCACAAATC	3341–3371(T → K)
HN3356F	CACCAAAAGACCAA**GTC**AATGTGCTGGACC	3356–3385(I → V)
HN3356R	GGTCCAGCACATT**GAC**TTGGTCTTTTGGTG	3356–3385(I → V)
ZP1F	CAACCTACACTTCATGTTCACAGG	2883–2906
ZP2R	AGACGGTCGCTACAACGGAAGT**AGT**AAG	3610–3659(C → S,S → G,A → T,
	TAC**GGT**GAC**ACCAGCACGAAC**	R → S,V → T,S → N)
ZP3F	**GTTCGTGCTGGT**GTC**ACC**GTACTT**ACT**A	3610–3659(C → S,S → G,A → T,
	CTTCCGTTGTAGCGACCGTCT	R → S,V → T,S → N)
ZP4R	TGCTTGTGTCTAGCGTCACTCG	3815–3836

Mutated nucleotides are underlined and in bold and the codon of the mutation effects are indicated in bold. Amino acid changes are shown in brackets.

### Recovery of viruses

The full-length cDNA clone and its derivative were linearized by digestion with *Not* I and purified using QIAquick PCR Purification Kit (Qiagen). Confluent BSR-T7/5 cells (4–6 × 10^6^ in a six-well plate) were separately transfected with mixtures of 2.5 μg linearized plasmid DNAs and 15 μl Lipofectamine^TM^ 2000 (Invitrogen) in a total volume of 1 ml OptiMEM. After 5 h at 37°C, 1 ml of GMEM supplemented with 4% tryptose phosphate broth and 10% FBS was added, and incubation continued for 2 to 3 days at 37°C. The supernatants were collected when CPE appeared. Each supernatant was then serially passaged for further experiments. The total RNA was extracted from passage 4 supernatant of each rescued virus and analyzed by RT-PCR followed by sequencing to verify that the rescued viruses were derived from the cDNA plasmids. The rescued viruses generated from the full-length plasmids pOFS and pOFSM were then designted as r-HN and rM-HN, respectively.

### Replication kinetics of rescued FMDV

To determine the replication kinetics of wild virus and rescued viruses in more detail, one-step growth was analyzed. BHK-21 cell monolayers were infected with wild O/HN/CHA/93 virus as well as two rescued viruses at a MOI of 0.1 TCID_50_ per cell. The cells were washed at 1 h postinfection, and then incubated at 37°C for 4, 8, 12 and 16 h. After incubation, infected cell cultures were harvested at 4 h intervals and the titer of infectious progeny was determined with TCID_50_ per milliliter using Reed–Muench formula.

### Stability of the genetically modified virus

Progeny viruses obtained from the full-length plasmids were serially passaged 10 times in BHK-21 cells. Viral RNA was extracted from virus stocks collected at passages 5 and 10. P1 coding region was amplified by RT-PCR and analyze to evaluate the genetic stability of the recombinant viruses.

### Serological cross-reactivity

Swine serums against O/HN/CHA/93 and rM-HN viruses were prepared by 28 days post vaccination using BEI-inactivated 146 S particles. O/Tibet/CHA/99, O/TAW/TL/97 and O/JX/CHA/2010 isolates were assessed for their serological relationship (r-value) to the O/HN/CHA/93 and rM-HN in a two-dimensional neutralisation test as described elsewhere [31].

### Swine immunization and challenge

A total of 110 six-week-old pigs were randomly allocated to nine groups. Six groups (groups 1–6) consisted of 16 pigs each; whereas three groups of 2 pigs each (groups 7–9) were used as control groups. All animals were sero-negative for FMDV 3ABC non-structural protein (NSP) antibodies prior to experimental vaccination. Two water-in-oil-in-water (WOW) vaccines were prepared from O/HN/CHA/93 and rM-HN to contain 2 μg of BEI-inactivated, sucrose density gradient purified 146 S FMDV antigen per 2 ml dose. The inactivated FMDV antigen was emulsified with Montanide ISA 206 (Seppic, France) oil. Groups 1, 3, and 5 were vaccinated with 2 ml vaccine containing inactivated O/HN/CHA/93 virus as antigen. Subsequently, groups 2, 4, and 6 were vaccinated with 2 ml vaccine containing inactivated rM-HN virus as antigen, whereas groups 7, 8, and 9 were inoculated with minimal essential medium as non-vaccinated. At 28 days post vaccination (dpv), all pigs were challenged intramuscularly with 10^5^ TCID50/2 ml of different FMDV at the ear-root-neck area. Briefly, groups 1, 2, and 7 were challenged with O/Tibet/CHA/99 virus. Groups 3, 4, and 8 were challenged with O/TAW/TL/97 virus. Groups 5, 6, and 9 were challenged with O/JX/CHA/2010 virus. Serum samples were collected from all animals on 0 and 28 dpv for virus neutralization titer analysis as described previously [32]. The neutralizing antibody titers were calculated as the log_10_ of the reciprocal antibody dilution required for 50% neutralization of 100 TCID_50_ viruses. The animals were then observed for the appearance of clinical signs of FMD daily for 14 days post challenge. Lesions were defined as described previously [33]. Briefly, localized lesions: vesicles observed on snout, lips, or one foot during the post-challenge period; generalized lesions: vesicles observed on snout, lips, and one or more feet during the post-challenge period. Two-week post-challenge, all animals were rebled, and sera were tested for the presence of antibodies to the FMDV non-structural proteins (NSP) 3ABC, using the commercially available 3ABC-I-ELISA kit. Samples were considered positive if the cutoff value ≥0.2. All animal studies were approved by the Review Board of Lanzhou Veterinary Research Institute, Chinese Academy of Agricultural Sciences (Permission number: SYXK-GAN-2004-0005). All animals used in the present study were humanely bred to bleed during the experiment and euthanasia was carried out at the end of the experiment.

## Results

### Amino acid variation of FMDV isolates of CHY topotype

Deduced amino acid sequence alignments were performed with the data for each isolates of CHY topotype using SeqMan II. The results indicated that numerous amino acid replacements occurred in the P1 or VP1 capsid region of the isolates of CHY topotype. These viruses shared consensus amino acids substitutions at antigenic site 1 (134 C → S, 137 S → G, 139 A → T, 140 R → S, 141 V → T, 142 S → N), 3 (43 T → K, 48 I → V), and 4 (58 E → D), compared with the O/HN/CHA/93 vaccine strain. The variations in the antigenic sites may render the available vaccine ineffective. The differences of amino acid sequences of FMDV isolates were showed in Figure 1.

### Generation of viruses

FMDV full-length cDNA clone, pOFS, was constructed in the M-pSK vector from the O/HN/CHA/93 vaccine strain. A genetically modified full-length clone, pOFSM, was assembled using overlap PCR fusion and Site-Directed Mutagenesis, based on the established infectious clone. The modified construct had nine amino acid differences (VP1 43 T → K, 48 I → V, 134 C → S, 137 S → G, 139 A → T, 140 R → S, 142 V → T, 142 S → N, VP3 58 E → D ) by sequence analysis, compared with the pOFS clone. Linearized pOFS and pOFSM plasmids were transfected into BSR-T7/5 cells to verify whether cDNA clones were infectious. In day 2 post-transfection, CPE appeared in both clones, indicating that the two full-length clones were infectious. Furthermore, some amino acid substitutions in the vaccine strain genome did not have an effect on the ability of viral replication in vitro.

RT-PCR was performed to amplify targeted fragments including genetic tags or targeted amino acid substitutions from viral RNAs of passage 4 to verify that the two rescued viruses were of recombinant origin. The result of sequence analysis confirmed that the recovered viruses were indeed derived from the respective recombinant plasmids.

### Replication kinetics of rescued FMDV

**Figure 3:**
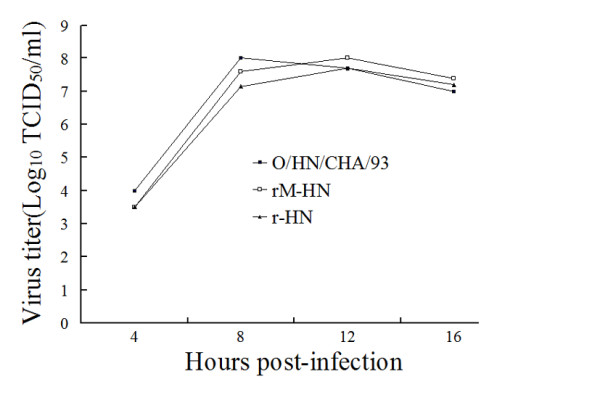
**Single-step growth curves of wild virus and recombinant viruses.** BHK-21 cells were infected with wild virus and recombinant viruses at MOI of 0.1 TCID_50_ per cell. At several time points, cells were harvested and the titers of the viruses were determined with TCID_50_ per milliliter using Reed–Muench formula.

Single-step growth curves of the rescued viruses and the wild virus were performed on BHK-21 cells. The results showed that the rescued viruses had the similar replication properties to the parental virus on BHK-21 cells (Figure 3). These results suggest that the substitutions of amino acid in capsid protein do not affect the ability of viral replication in vitro.

### Stability of the genetically modified FMDV

The recombinant r-HN and rM-HN viruses were serially passaged 10 times in BHK-21 cells. Consequently, the amino acid sequence of P1 coding regions of the recombinant viruses recovered from passages 5 and 10 were analyzed. The results revealed that the rescued viruses remained genetically stable during 10 serial passages in vitro.

### Serological cross-reactivity

The relative homology value (r1) between field isolate (O/Tibet/CHA/99, O/TAW/TL/97, and O/JX/CHA/2010) and reference strains (O/HN/CHA/93 and rM-HN) were determined using two dimensional micro neutralization tests (Table 4) as previously described [34]. The results showed that the O/HN/CHA/93 reference strains have close antigenic relationship with O/Tibet/CHA/99 and O/JX/CHA/2010 isolates, but the O/TAW/TL/97 isolate is antigenically related to the O/HN/CHA/93 vaccine strain, which indicates that a more potent vaccine strain needs to be developed against the variants of the CHY topotype. However, the rM-HN virus has close antigenic relationship with isolates of three topotypes compared with the wild O/HN/CHA/93 virus. Therefore, this virus would be used as a vaccine candidate.

**Table 4:** The “r” values obtained between field isolates and reference strains

**Virus**	**O/HN/CHA/93**	**rM-HN**
O/Tibet/CHA/99	0.57	0.60
O/TAW/TL/97	0.28	0.45
O/JX/CHA/2010	0.59	0.54

### Swine protection experiments

To test the protective efficacy of the inactivated oil-emulsified vaccines that were prepared from wild O/HN/CHA/93 strain and the genetically modified virus rM-HN against the viruses of three topotypes, the present study designed a swine vaccination and challenge studies. All pigs immunized with vaccine prepared from rM-HN were fully protected from clinical disease and viral infection after challenge with O/Tibet/CHA/99, O/TAW/TL/97, and O/JX/CHA/2010 viruses. No clinical signs of FMD were detected in these vaccinated pigs. The pigs vaccinated with O/HN/CHA/93 vaccine were completely protected against O/Tibet/CHA/99 and O/JX/CHA/2010 virus infection. However, 4 of the 16 pigs developed localized lesions after O/TAW/TL/97 virus infection, although these animals had a delayed appearance of vesicles (4–5 dpc) compared with the control swine. All control animals in groups 7, 8, and 9 developed generalized FMD and clinical signs of FMD appeared 2 to 3 days after the infection of these viruses. All immunized pigs developed a higher neutralizing antibody against these viruses, except for the immunized pigs that produced clinical signs of FMD. The present study indicated that vaccine prepared from genetically modified FMDV by reverse genetics significantly improved protective efficacy to the variant of the CHY topotype, compared with the O/HN/CHA/93 strain. Tables 5, 6, and 7 present the results of swine protection experiments. Two weeks post-challenge, animals were rebled and sera were tested for the presence of antibodies to the nonstructural protein 3ABC. As expected, antibodies against NSP 3ABC could be detected in un-vaccinated controls and in the four immunized pigs that produced clinical signs of FMD after 14 days post-challenge. Whereas all of the other vaccinated pigs remained NSP seronegative (Tables 5, 6, and 7).

**Table 5:** Responses of pigs to vaccination with O/HN/CHA/93 and rM-HN vaccines and O/Tibet/CHA/99 challenge

**Vaccine^a^**	**Pig no.**	**VNT titer versus O/Tibet/CHA/99^b^**			**Vaccine^a^**	**Pig no.**	**VNT titer versus O/Tibet/CHA/99^b^**		
			**Lesions^c^**	**3ABC^d^**				**Lesions^c^**	**3ABC^d^**
O/HN/CHA/93	101	1.6	-	-	rM-HN	601	2.1	-	-
	102	1.7	-	-		202	1.6	-	-
	103	2.0	-	-		203	1.6	-	-
	104	1.7	-	-		204	2.0	-	-
	105	1.5	-	-		205	1.8	-	-
	106	2.1	-	-		206	2.1	-	-
	107	1.8	-	-		207	1.7	-	-
	108	2.0	-	-		208	1.9	-	-
	109	1.5	-	-		209	1.8	-	-
	110	1.9	-	-		210	2.0	-	-
	111	2.1	-	-		211	1.8	-	+
	112	1.9	-	-		212	2.0	-	-
	113	1.8	-	-		213	1.6	-	-
	114	1.6	-	-		214	2.1	-	-
	115	2.1	-	-		215	1.7	-	-
	116	1.7	-	-		216	2.0	-	-
Control	117	<1.0	++	+	Control	217	<1.0	++	+
	118	<1.0	++	+		218	<1.0	++	+

^a^ BEI-inactivated virus.

^b^ Day 28 virus neutralization titer (expressed as the log_10_ of the reciprocal antibody dilution required for 50% neutralization of 100 TCID_50_ virus) values represent the means from two repeat tests.

^c^ Scoring of clinical signs: -, no visible lesions, +, localized gross lesions; + +, generalized lesions.

^d^ Presence of antibodies to the nonstructural protein 3ABC in sera collected 14 days post-challenge, determined by the commercially available 3ABC-I-ELISA kit. Samples were considered positive if the cutoff value ≥0.2.

**Table 6:** Responses of pigs to vaccination with O/HN/CHA/93 and rM-HN vaccines and O/TAW/TL/97 challenge

**Vaccine^a^**	**Pig no.**	**VNT titer versus O/TAW/TL/97^b^**			**Vaccine^a^**	**Pig no.**	**VNT titer versus O/TAW/TL/97^b^**		
			**Lesions^c^**	**3ABC^d^**				**Lesions^c^**	**3ABC^d^**
O/HN/CHA/93	301	1.8	-	-	rM-HN	401	2.1	-	-
	302	1.5	-	-		402	1.6	-	-
	303	1.7	-	+		403	1.6	-	-
	304	1.5	-	-		404	1.9	-	-
	305	1.6	-	-		405	1.8	-	-
	306	1.7	-	-		406	2.1	-	-
	307	0.7	+	+		407	1.7	-	-
	308	1.5	-	-		408	1.6	-	-
	309	1.6	-	-		409	1.8	-	-
	310	0.8	+	+		410	2	-	-
	311	1.8	-	-		411	1.8	-	-
	312	1.0	+	+		412	1.7	-	-
	313	1.5	-	-		413	1.6	-	-
	314	1.6	-	-		414	2.1	-	-
	315	1.7	-	-		415	1.7	-	-
	316	0.8	+	+		416	1.6	-	-
Control	317	<1.0	++	+	Control	417	<1.0	++	+
	318	<1.0	++	+		418	<1.0	++	+

^a, b, c, d^ described as above.

**Table 7:** Responses of pigs to vaccination with O/HN/CHA/93 and rM-HN vaccines and O/JX/CHA/2010 challenge

**Vaccine^a^**	**Pig no.**	**VNT titer versus O/JX/CHA/2010^b^**			**Vaccine^a^**	**Pig no.**	**VNT titer versus O/JX/CHA/2010^b^**		
			**Lesions^c^**	**3ABC^d^**				**Lesions^c^**	**3ABC^d^**
O/HN/CHA/93	501	1.6	-	-	rM-HN	601	1.9	-	-
	502	2.3	-	-		602	2.1	-	-
	503	1.7	-	-		603	1.6	-	-
	504	2.0	-	-		604	1.7	-	+
	505	1.9	-	-		605	2.0	-	-
	506	2.0	-	-		606	1.8	-	-
	507	2.2	-	-		607	2.0	-	-
	508	1.7	-	+		608	2.1	-	-
	509	2.1	-	-		609	1.7	-	-
	510	1.8	-	-		610	2.0	-	-
	511	2.1	-	-		611	1.7	-	-
	512	1.9	-	-		612	2.0	-	-
	513	1.6	-	-		613	1.9	-	-
	514	1.8	-	-		614	1.8	-	-
	515	1.6	-	-		615	1.7	-	-
	516	2.2	-	-		616	1.7	-	-
Control	517	<1.0	++	+	Control	617	<1.0	++	+
	518	<1.0	++	+		618	<1.0	++	+

^a,b,c,d.^ described as above.

## Discussion

FMD remains one of the most economically important diseases of farm animals and is widespread across the world, especially in Africa, Asia and South America. Type O FMD is prevalent in China (including Chinese Taipei, Chinese Hong Kong) and its surrounding countries [10-13]. Among the epidemic topotypes in these regions, the viruses in CHY topotype are highly adapted to pigs [15], which represents the biggest threat on the Chinese hog industry and the economy. For instance, in 1997, a devastating and unusual outbreak of FMD occurred in Taiwan. The outbreak, which was caused by a CHY topotype virus, rapidly developed into massive epizootic, resulting in the slaughter of more than 4 million pigs and financial losses of over 6 billion U.S. dollars [35,36]. China is the biggest pork producer and consumer in the world and the pig industry has become the most important sector in Chinese animal husbandry [37]. Therefore, control of FMD in swine has become high priority. Vaccination as a control tool has been gaining favor as a potentially more effective approach for controlling the virus, reducing the economic loss to animal husbandry, and contributing to improved food security in China. However, currently available FMD vaccines in China often do not provide complete protection against some variants arising in the CHY topotype.

Extensive studies has showed that the G–H loop of FMDV is the major immunodominant site [38,39], and it can induce a strong antibody response against the virus, which is known to play a major role in protection induced by the current FMDV vaccines [9]. Experimental peptide or recombinant vaccines [40,41] have been based mainly on this major antigenic site of FMDV. In addition, FMDV antigenic variation also occurs within other antigenic sites, which are implicated in the full complete immunologic response [9,42]. Based on these theories, the present study compared amino acid sequences of the capsid region of O/HN/CHA/93 vaccine strain and 18 reference isolates of CHY topotype. The results revealed that consensus amino acid substitutions occurred at VP1 (43 T → K, 48 I → V 134 C → S, 137 S → G, 139 A → T, 140 R → S, 141 V → T, 142 S → N) and VP3 (58 E → D), which are involved in antigenic site 1, 3, and 4. Among these substitutions, six were located at the G-H loop of VP1 and three were located at a position critical for antigenic sites 3 and 4. Any alteration of critical residues would confer antigenic specificity to the FMD viral variants [43-45]. Therefore, we presumed that the antigenic differences between the variants of CHY topotype and the current vaccine strain might account for incomplete protection.

Previous successes in the generation of engineered avirulent, chimeric, and thermostable FMDV vaccine candidates [33,46,47] have further provided insight for designing new vaccine candidate viruses with engineered modifications by reverse genetics. Therefore, the present study constructed a full-length infectious clone of O/HN/CHA/93 vaccine strain and created a genetically modified construct with amino acid substitutions (1 C 58 E → D, 1D 43 T → K, 48 I → V, 134 C → S, 137 S → G, 139 A → T, 140 R → S, 141 V → T, 142 S → N), based on vaccine strain framework using a similar strategy. As expected, a genetically modified virus was obtained from the modified full-length plasmid. Specifically, amino acid substitutions in the capsid protein did not affect the in vitro infectivity properties of the recombinant. Viability of the virus indicated that the genome of FMDV O/HN/CHA/93 can tolerate these amino acid replacements at three antigenic sites. This finding was not surprising, because previous studies have demonstrated that FMDV can accommodate replacements of G-H loop, capsid coding region as well as other gene region of inter-genotypic and intra-genotypic [16,42,48-50]. The recombinant viruses were genetically stable after 10 serial passages in BHK-21 cells.

As a FMD vaccine candidate, the virus should necessarily grow to high yield in tissue culture for sufficient antigenic mass to be produced [51]. Therefore, the replicative properties of the recombinant viruses were assessed by single-step growth curves. The results indicated that the rescued viruses had similar growth properties to the wild O/HN/CHA/93 virus. In addition, an ideal vaccine candidate would induce cross protection against viruses from different antigenic groups within the subtype. Analysis of antigenic relationships between the field isolate of the three topotypes and the reference strains (O/HN/CHA/93 and rM-HN) showed that the O/HN/CHA/93 reference strains demonstrated close antigenic relationship with O/Tibet/CHA/99 and O/JX/CHA/2010 isolates. However, the O/TAW/TL/97 isolate is antigenically related to the vaccine strain. The rM-HN virus had close antigenic relationship with isolates of the three topotypes compared with the O/HN/CHA/93 strain. Thus, this recombinant virus would be used as vaccine candidate. Comparative efficacy of the inactivated vaccines prepared from the rM-HN and O/HN/CHA/93 viruses were tested in swine. The results demonstrated that pigs vaccinated with the O/HN/CHA/93 vaccine were fully protected from O/Tibet/CHA/99 and O/JX/CHA/2010 virus challenge, but only 75% of the immunized animals were against O/TAW/TL/97 infection. However, all pigs vaccinated with the rM-HN vaccine obtained complete protection against O/Tibet/CHA/99, O/TAW/TL/97, and O/JX/CHA/2010 virus challenge, which may be contribute to the more antigentic similarities of the genetically modified virus with the isolate of CHY topotype.

## Conclusions

The present study constructed a full-length infectious cDNA clone of FMDV vaccine strain and created a genetically modified virus with some amino acid substitutions in capsid protein, based on vaccine framework. Some amino acid substitutions in the FMDV vaccine strain genome did not have an effect on the ability of viral replication in vitro. The recombinant viruses had similar growth properties with the wild O/HN/CHA/93 virus. The vaccine prepared from genetically modified FMDV significantly improved protective efficacy to the variants of the CHY topotype, compared with the wild O/HN/CHA/93 virus. Thus, the full-length cDNA clone of FMDV can be a useful tool to develop genetically engineered FMDV vaccine candidates to help control porcinophilic FMD epidemics in China. Furthermore, the present study provided further insights for designing genetically modified FMD vaccine candidate by reverse genetics, based on the sequence information of arising mutants during the new FMD outbreak.

## Competing interests

The authors declare that they have no competing interests.

## Authors’ contributions

PHL conceived and designed the study. XWB constructed the FMDV full-length infectious cDNA clone and modified construct. PS and DL carried out the animal experiments. ZJL performed sequence alignment. YMC and YFF performed virus neutralization assay. HFB, YLC and BXX passaged the recombinant viruses. PS contributed reagents, materials, and analysis tools. ZXL supervised all aspects of the research. PHL analyzed the data and drafted the manuscript. All authors read and approved the final manuscript.
